# Indicators of the need for ICU admission following suicide bombing attacks

**DOI:** 10.1186/1757-7241-20-19

**Published:** 2012-03-09

**Authors:** Miklosh Bala, Dafna Willner, Asaf Keidar, Avraham I Rivkind, Tali Bdolah-Abram, Gidon Almogy

**Affiliations:** 1 Department of General Surgery and Trauma Unit, Hadassah-Hebrew University Medical Centre, Jerusalem, Israel; 2 Department of Anaesthesiology and Critical Care Medicine, Hadassah-Hebrew University Medical Centre, Jerusalem, Israel; 3 Hadassah School of Medicine, Hadassah-Hebrew University Medical Centre, Jerusalem, Israel

**Keywords:** Blast injury, Suicidal attacks, Intensive care unit, Triage

## Abstract

**Introduction:**

Critical hospital resources, especially the demand for ICU beds, are usually limited following mass casualty incidents such as suicide bombing attacks (SBA). Our primary objective was to identify easily diagnosed external signs of injury that will serve as indicators of the need for ICU admission. Our secondary objective was to analyze under- and over-triage following suicidal bombing attacks.

**Methods:**

A database was collected prospectively from patients who were admitted to Hadassah University Hospital Level I Trauma Centre, Jerusalem, Israel from August 2001-August 2005 following a SBA. One hundred and sixty four victims of 17 suicide bombing attacks were divided into two groups according to ICU and non-ICU admission.

**Results:**

There were 86 patients in the ICU group (52.4%) and 78 patients in the non-ICU group (47.6%). Patients in the ICU group required significantly more operating room time compared with patients in the non-ICU group (59.3% vs. 25.6%, respectively, *p *= 0.0003). For the ICU group, median ICU stay was 4 days (IQR 2 to 8.25 days). On multivariable analysis only the presence of facial fractures (*p *= 0.014), peripheral vascular injury (*p *= 0.015), injury ≥ 4 body areas (*p *= 0.002) and skull fractures (*p *= 0.017) were found to be independent predictors of the need for ICU admission. Sixteen survivors (19.5%) in the ICU group were admitted to the ICU for one day only (ICU-LOS = 1) and were defined as over-triaged. Median ISS for this group was significantly lower compared with patients who were admitted to the ICU for > 1 day (ICU-LOS > 1). This group of over-triaged patients could not be distinguished from the other ICU patients based on external signs of trauma. None of the patients in the non-ICU group were subsequently transferred to the ICU.

**Conclusions:**

Our results show that following SBA, injury to ≥ 4 areas, and certain types of injuries such as facial and skull fractures, and peripheral vascular injury, can serve as surrogates of severe trauma and the need for ICU admission. Over-triage rates following SBA can be limited by a concerted, focused plan implemented by dedicated personnel and by the liberal utilization of imaging studies.

## Background

We are currently witnessing suicidal attacks in several countries against diverse populations using violence or the threat of violence to attain political, religious, or ideological goals resulting in physical harm and material damages, and the spread of fear and panic [1]. Explosive devices activated by suicide attackers have emerged as the new lethal weapon of terrorist organizations [2]. Suicide bombing attacks (SBA) present unique triage, diagnostic, and management challenges. To optimize the treatment of mass casualties and the chaos that ensues, victims need to be directed to the appropriate level of care, and life-threatening injuries need to be swiftly recognized and treated. In these circumstances early diagnosis depends on a well-functioning trauma system, adequate logistics, sufficient training, a focused physical examination and use of targeted imaging studies.

Victims of SBA will typically suffer a combination of blast injury, penetrating missiles, blunt trauma and burns. Intentional explosions cause more severe injuries (Injury Severity Score [ISS] ≥ 16) and require more abdominal, vascular and neurosurgical procedures compared with other forms of trauma [3,4]. Several studies have shown that critical hospital resources, particularly the demand for intensive care unit (ICU) beds, are usually severely stressed following a SBA [5,6]. A third of ICU admissions were directly from the emergency department (ED), and 12% of patients bound for the ICU were admitted to a post anaesthesia care unit (PACU) bed due to ICU overflow [7].

Jerusalem was the target of 17 of the 59 suicide bombing attacks that took place in Israel from August 2001 to August 2005 [8]. The Hadassah University Hospital Ein-Kerem Campus (HUH) is the only level I trauma centre in Jerusalem. We have previously evaluated the experience acquired at our hospital in managing and treating survivors of SBA [9,10]. Victims with abnormal vital signs are obviously severely injured and will be treated in a trauma unit setting. Victims who walked into the ER with normal vital signs have been reported to collapse within minutes. Recognition of these injuries in the context of mass casualties must be performed rapidly. The primary objective of this study was to identify external signs of trauma and readily diagnosed types of injuries that will function as indicators for ICU admission in victims of SBA. The secondary objective was to quantify over- and under-triage under these circumstances and predict which victims were over- or under-triaged.

## Methods

We retrospectively reviewed the records of all victims of suicide bombing attacks who were admitted to HUH, in Jerusalem between August 2001 and August 2005. Data was retrieved from medical records and the trauma registry database. The trauma registry is a prospectively collected database that is updated daily by dedicated personnel and has institutional review board (IRB) approval. The charts of all patients admitted to the hospital with the diagnosis of trauma are reviewed and demographic data such as gender, age, cause of trauma, and types of injuries and their location and severity are recorded. The registry is updated daily for diagnostic and surgical procedures that were performed and patient outcome. ISS is calculated based on the Abbreviated Injury Scale 2005 edition [11].

Surgical ICU beds available at HUH are the general surgery ICU, neurosurgical ICU, cardio-thoracic ICU and the PACU beds transformed into ICU beds per demand. PACU beds, in an emergency setting, can be converted to additional ICU beds [7].

The protocol at HUH following a mass casualty incident was previously described [9]. Briefly, each admitted patient is normally delegated a resident surgeon and an anaesthesiologist/ICU resident who accompanies the patient throughout initial evaluation and into the operating room, if required [9]. Every 3-4 patients are assigned an attending surgeon and ICU staff who supervise the process of triage, the initial evaluation and treatment.

### Data collection

The trauma registry database was screened for patients who had physical injuries and were admitted to the hospital following a SBA. Data was collected from medical charts and the trauma registry for demographic characteristics, the presence and location of penetrating wounds, the location (extremity vs. skull and facial) and type of fractures (open vs. closed), the presence of burns, ear-drum perforation, blast lung injury (BLI), and ISS.

### Data analysis

Patients were divided into two groups: those initially admitted to an ICU (ICU group) and those patients admitted to a ward (non-ICU group). Patients who were admitted to the recovery room for less than 24 hours following a surgical procedure were included in the non-ICU group.

The body was divided into nine areas (head, neck, cervical spine, chest, upper back, abdomen, lower back, legs and arms) and the presence of a visible injury to each area, such as a penetrating wound or burn, was retrieved from the trauma registry database [12]. To quantify the extent of external injury we added the sum of injured areas and defined it as the "number of areas injured" (range of 1 to 9). We used the cut-off of ≥ 4 body areas injured and defined it as "injury to multiple areas". To analyze the distribution and importance of a penetrating injury, the nine areas of the body were grouped into three zones: head (head, neck and cervical spine), torso (chest, abdomen, and upper and lower back) and extremities (legs and arms).

We defined an ICU stay of less than 24 hours as over-triage. Patients who died on the day of admission (early deaths) from uncontrollable haemorrhage (4 patients) in the operating room were excluded from this group. Under-triage was defined as when a patient required admission to the ICU after being initially triaged to a ward and not as a result of a surgical complication or a death that was caused by primary patient management in an improper setting.

### Statistical analysis

Data was presented as median and interquartile range (IQR). Fisher's exact test was used to compare proportions and the Mann-Whitney *U *test was used to compare continuous variables. Multivariable logistic regression analysis was performed to analyze predictors of the need for ICU admission. Parameters entered into the regression model were burns, injury to 4 or more body areas, penetrating torso injury, peripheral vascular injury, skull, facial, and open fractures,. A p value of 0.05 or less was considered statistically significant. Statistical analysis was performed using SPSS version 18.0 (Statistical Package for Social Science, Chicago, Ill).

## Results

### Patient population

From August 2001 to August 2005 there were 17 suicide bombing attacks in Jerusalem. 430 patients were examined in the Emergency Department of HUH and 167 patients (38.8%) were admitted. Three patients who were transferred directly from other hospitals to an ICU were excluded from the study. There were 86 patients in the ICU group (52.4%) and 78 patients in the non-ICU group (47.6%) (Table 1).

**Table 1:** Demographic and clinical characteristics of patients in the ICU and non-ICU groups

	ICU Group (n = 86)	Non-ICU Group (n = 78)	*P *value
Age (yrs)*	26 (18.25-35.75)	24 (17.25-34)	1.0†

Gender (males) (%)	44 (51.2)	40 (51.3)	1.0‡

Attack setting (bus, %)	43 (50)	34 (43.6)	0.437‡

LOS (days)*	13 (8-22)	5.5 (3-9)	0.0001†

Trauma Unit (%)	69 (80.2)	9 (11.5)	0.0001‡

Operating room (%)	51 (59.3)	20 (25.6)	0.0001‡

Data shown as number (and percent) or median* (and IQR); † Mann-Whitney *U *test; ‡ Fisher's exact test

More than three quarters of the patients in the ICU group were initially triaged to the Trauma Unit by the surgeon-in-charge compared with only 11.5% of patients in the non-ICU group (*p *< 0.0001). Patients in the ICU group also required more surgical intervention (i.e. operating room) compared with patients in the non-ICU group (51 patients [59.3%] vs. 20 patients [25.6%], respectively, *p *= 0.0003) (Table 1). Median stay in the ICU was 4 days (IQR 2 to 8.25 days).

### Types of injuries

Univariate analysis of the common types of injuries in both groups is shown in Table 2. Injury to 4 or more body regions was significantly more common in the ICU group. Interestingly, the rate of ear drum perforation was not different between the groups. Injury to the extremities including all types of penetrating extremity injury and open extremity fractures were not different between the two groups. Torso fractures including skull and facial fractures and rib fractures were significantly more common in the ICU group (Table 2).

**Table 2:** Univariate analysis of the types of injury in the ICU and non-ICU groups

	ICU Group (n = 86)	Non-ICU Group (n = 78)	*P *value*
Injury to 4 or more regions	38 (44.2)	11 (14.1)	< 0.0001

Burns	31 (36)	15 (19.2)	0.0245

Ear drum perforation	20 (23.3)	16 (20.5)	0.709

Open extremity fractures	22 (25.6)	13 (16.7)	0.185

Peripheral vascular injury	14 (16.3)	3 (3.8)	0.01

Skull fractures	18 (20.9)	5 (6.4)	0.012

Facial fractures	17 (19.8)	1 (1.3)	< 0.0001

Rib fractures	12 (13.9)	0 (0)	0.004

Penetrating extremity injury	41 (47.7)	38 (48.7)	1.0

Penetrating torso injury	48 (55.8)	21 (26.9)	0.0002

Penetrating head injury	60 (69.8)	32 (41)	0.0003

Data shown as number and (percent); * Fisher's exact test

### Under-triage

The number of patients who were under-triaged was evaluated by assessing the number of patients who were initially admitted to the floor or to the operating room (non-ICU group) and later transferred to the ICU because of clinical deterioration, and by the number of deaths who could have been prevented if initially admitted to an ICU.

None of the patients (0%) in the non-ICU group were transferred later to the ICU. There were 10 in-hospital deaths: five patients died from severe intracranial injury (median 8 days after admission). Two patients died from penetrating intra-thoracic injury (both on the day of admission), and two patients died from uncontrollable retroperitoneal and intra-abdominal haemorrhage (both on the day of admission); all four died despite attempts at temporizing the situation. One patient died from severe BLI (day 4). All ten patients (100%) were either admitted to the ICU or taken directly to the operating room, and were treated in the ICU throughout the rest of their hospital course. None of the deaths could be attributed to under-triage.

### Over-triage

**Figure 1:**
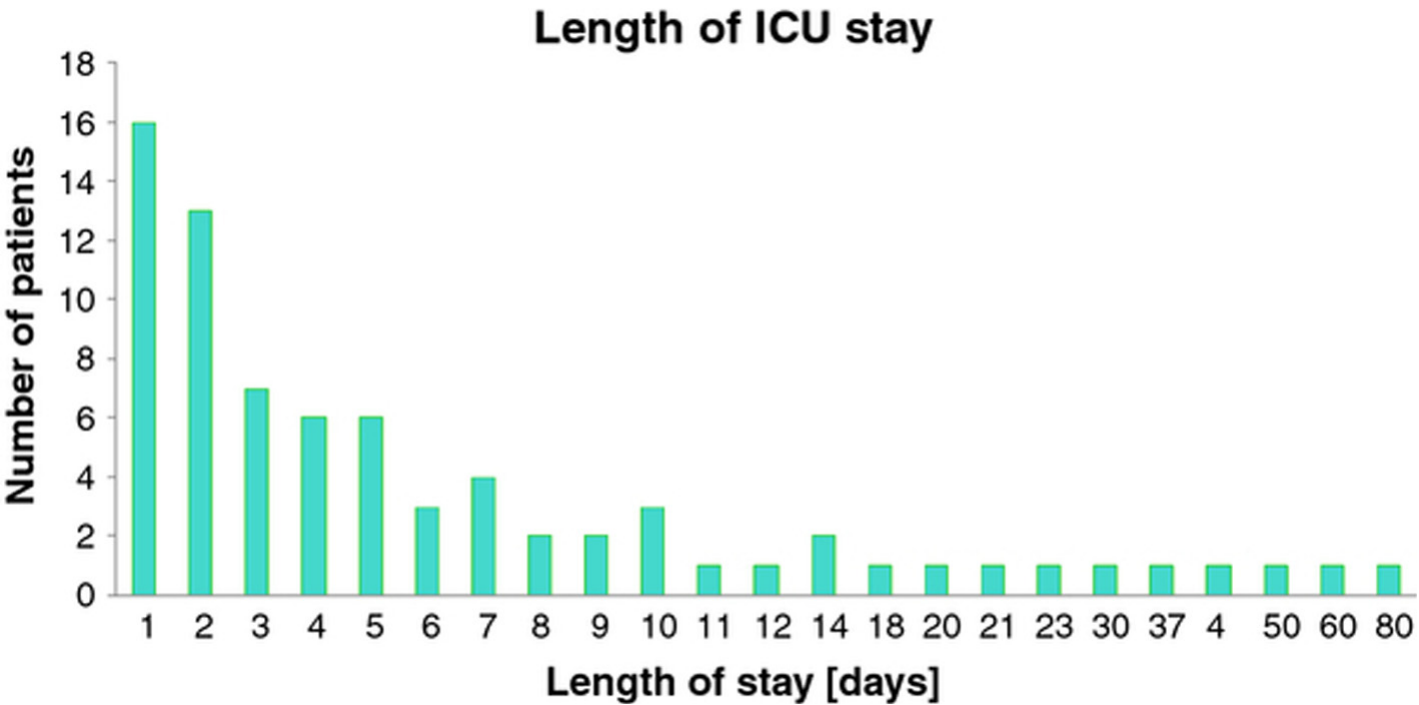
**Length of stay in the ICU along time (note that time axis is not linear)**.

Median ICU length of stay (LOS) was 4 days (IQR 2 to 8.25 days) and median hospital LOS for the ICU group was 13 days (IQR 8.75 to 27.5 days) (Figure 1). Sixteen patients (19.5%) in the ICU group were admitted to the ICU for one day only (excluding the 4 patients who died on the day of admission from uncontrollable haemorrhage) (ICU LOS = 1). The different types of injuries among the ICU groups are shown in Table 3. Penetrating torso injuries were more common in the ICU LOS > 1 group compared with the ICU LOS = 1 group but the differences were not significant (35 patients [56.5%] vs. 5 patients [31.3%], respectively, *p *= 0.095) (Table 3).

**Table 3:** Univariate analysis of the types of injury in the ICU group according to the number of days in the ICU

	ICU LOS = 1 (n = 16)	ICU LOS > 1 (n = 66)	*P *value*
Median ISS (IQR)	14.5 (9-17)	25 (16-34)	0.0001†

Injury to 4 or more regions	6 (37.5)	30 (45.5)	0.78

Intubated in ED	6 (37.5)	38 (57.6)	0.404

Burns	6 (37.5)	24 (36.4)	1.0

Ear drum perforation	4 (25)	15 (22.7)	1.0

Open extremity fractures	3 (18.8)	19 (28.8)	0.539

Peripheral vascular injury	2 (12.5)	11 (16.7)	1.0

Skull fractures	1(6.3)	15 (22.7)	0.176

Facial fractures	2 (12.5)	15 (22.7)	0.503

Rib fractures	2 (12.5)	9 (13.6)	1.0

Penetrating extremity injury	7 (43.8)	32 (48.5)	0.786

Penetrating head injury	9(56.3)	48 (72.7)	0.233

Penetrating torso injury	5 (31.3)	35 (59.1)	0.095

Data shown as number (and percent); * Fisher's exact test; † Mann-Whitney *U *test; Four patients who died on the day of admission from massive haemorrhage were excluded from this group (see text)

### Predictors of the need for ICU admission

Multivariable regression analysis was performed in order to identify predictors for ICU admission. Injuries that obviously mandated ICU admission such as severe intra-cranial injury, BLI and severe abdominal trauma were not entered into the model. External signs of trauma and injuries with clinical significance including penetrating torso injury, open fractures, skull and facial fractures, peripheral vascular injury, burns and injury to 4 or more areas were entered into a logistic regression model. The presence of facial fractures, peripheral vascular injury, injury to 4 or more areas, and skull fractures were found to be independent predictors of the need for ICU admission (Table 4).

**Table 4:** Multivariable analysis of predictors of the need for ICU admission

	Adjusted odds ratio	95% confidence interval	*P *value
Facial fractures	14.54	1.74-121.72	0.014

Peripheral vascular injury	6.21	1.42-27.14	0.015

Injury to 4 or more areas	4.16	1.67-10.34	0.002

Skull fractures	4.46	1.31-15.14	0.017

Penetrating torso injury	1.91	0.86-4.25	0.111

Open extremity fractures	0.96	0.366-2.52	0.934

Burns	1.83	0.48-7.0	0.377

## Discussion

Following a suicide bombing attack there is an influx of a large number of patients suffering from complex injuries over a short time period [9,13]. Victims suffer from a combination of blast injury, penetrating wounds and burns [3,13]. Triage performed at the scene and in the hospital will determine the distribution of casualties to the different medical and trauma centres, and the distribution of patients within a medical facility to the different levels of care, respectively. During this chaotic period, guidelines are necessary to improve triage, delivery of care and utilization of resources.

Several authors have previously shown the central role of ICU beds in the treatment of victims of suicide bombing attacks [14,15]. In fact, in countries where ICU bed availability is limited, it is considered by many to be the most severe bottleneck in the process of managing these unique circumstances. Aschkenasy-Steuer et al. reported on a median of 4 victims per attack (range 1 to 9) who were admitted to an ICU following a SBA [7]. As we and others have shown, the number of ICU admissions will depend on several factors such as the magnitude of the blast, attack setting (bus vs. open space) and the number of patients evacuated to the ED [5,7,16]. Our study demonstrates yet again the immense demand on ICU beds that develops following a SBA. More than one half of all patients (52.4%) admitted to the ED will require an ICU bed. The majority of these beds are not required immediately. Forty-two patients (48.8%) in the ICU group were transferred from the ED to the operating room and admitted to the ICU only following a surgical procedure. This allows time for ICU staff and hospital administration to discharge patients to the floor and transform PACU beds into additional ICU beds.

Penetrating injuries caused by flying debris and shrapnel are present in more than 85% of patients [12,17]. We have previously analyzed predictors for severe injuries requiring more urgent care such as severe BLI and intra-abdominal injury [18,19]. Our past experience has shown that among patients admitted to the ED, injury to four or more body regions, and the presence of penetrating head and torso injuries are predictors of BLI and intra-abdominal injury, respectively [16,20]. This current study confirms that injury to four or more body regions, an easily recognizable external sign of trauma, is associated with severe injury and foresees the need for ICU admission.

Injury from shrapnel depends on the distance of the victims from the blast's epicentre, the individual mass of the penetrating fragment, and the location of impact [21,22]. Our data shows that fractures to bony structures chiefly the head, is strongly associated with severe injury and with the need for ICU admission. Victims in proximity to the blast are more likely to suffer from injuries caused by heavy shrapnel that is often added to magnify the explosive effect. We believe that it is these heavy, high-energy particles that cause fractures to bony structures of the torso. These patients suffer from multiple wounds of various aetiology and perhaps these types of fractures (skull and facial) are surrogates to other types of severe injury which require an ICU admission.

Controversy exists regarding the significance of tympanic membrane (TM) rupture as a predictor of blast injury. In a recent review, the authors advocated the value of routine otoscopy in triaging victims of bombing attacks to identify those suffering from severe blast injury [17]. Leibovici et al. reported on 647 victims of 11 bombing attacks [16]. Of the 49 victims who suffered from BLI, 18 (36.7%) did not have a TM rupture. Our previous published results and our current data consistently show that TM rupture cannot be used to assess the severity of BLI and predict the need for ICU admission [23]. However, the relatively high rate of this injury (over 20% in our series) mandates ear drum examination following a SBA.

A "minimal work-up approach" has been encouraged following an MCI in order to facilitate the inflow of patients and prevent inundation of the admitting facility [24]. The hypothesis is to prevent the improper utilization of limited hospital resources on patients with severe injuries and a lower chance of survival. This practice following bombing attacks has lead to a high rate of negative procedures such as was reported following the London attacks (5 of 5 negative laparotomies, 100%) and the Madrid attacks (3 of 7 negative laparotomies (42.9%) [25,26]. Patients following negative laparotomy in these circumstances may require ICU admission, and this may shift patients towards over-triage. We have been successful in avoiding high over-triage rates (19.5% according to our data, compared to up to 50% in previous publications) and minimizing under-triage [27,28]. This is secondary to several key points. Primarily, each moderately to severely injured victim is attended to by a surgical resident and anaesthesiologist/ICU resident. The victim's situation is continuously reassessed by the surgeon-in-charge until a diagnosis is reached and a treatment plan outlined. We practice a liberal approach to the utilization of advanced imaging modalities [20]. All available manpower is activated by cancelling elective surgery. This approach lowers the rate of negative surgical explorations while maintaining under-triage at an acceptable level (0% in our experience).

The over-triaged group of 16 patients who spent only 1 day in the ICU could not be distinguished from the more severely injured patients based on physical findings and initial imaging studies. Thus, an over-triage rate of 19.5% (16 of 82 patients) is inevitable and should be expected in these circumstances. It is important to remember that the initial triage performed outside the ED cannot be perfect. Modern hospital triage, especially triage following an MCI, is an interactive process, with casualties being re-assessed again and again during their initial care to identify and correct earlier triage errors. As we previously published, moderately injured victims initially over-triaged to the trauma unit should be transferred to the admitting area of the ED following initial physical evaluation and imaging studies. Likewise, severely injured patients who were under-triaged to the admitting area should be transferred to the trauma unit or an ICU where their injuries can be treated more appropriately.

Seventeen patients (19.8%) in the ICU group were initially triaged to the admitting area. Many of them suffered from a penetrating injury to the head and torso, skull and/or facial fractures, and injury to 4 or more body areas. Following our results, patients with similar injuries or a combination of injuries, should probably be triaged to the trauma unit or ICU.

### Limitations

Data was retrospectively collected from the trauma registry. The trauma registry is compiled from patient charts and some injuries may not have been recorded, especially in a mass casualty setting when patients suffer from multiple wounds. Our definition of less than 24 hours admission to an ICU as a cut-off for over-triage is arbitrary and based on our clinical experience and does not necessarily reflect true over-triage. Nonetheless, and based on our unfortunate experience, we are confident our results offer a valid representation of the circumstances following a suicide bombing attack on a civilian population.

## Conclusions

Our results show that injury to 4 or more body areas, and specific types of injuries such as facial and skull fractures, and peripheral vascular injury, can serve as surrogates of severe trauma and the need for ICU admission. We recommend that the injury parameters we defined be incorporated into the protocols for trauma triage both at the scene and in the hospital.

## Competing interests

The authors declare that they have no competing interests.

## Authors' contributions

MB and DW coordinated data collection, participated in data analysis and drafted the manuscipt. AK participated in data analysis. AR supervised data collection and analysis. TBA assisted in statistical analysis. GA supervised data collection and data analysis, participated in statistical analysis and drafted the manuscript. All the authors read and approved the final manuscript.
